# Prevalence of needlestick injuries in dental assistants: a systematic review and meta-analysis

**DOI:** 10.7189/jogh.15.04030

**Published:** 2025-02-14

**Authors:** Jiang Zeng, Enhong Li, Yuxin Xu, Yuwei Lin, Yang Xiao, Xuefen Yu

**Affiliations:** 1 Center for Rehabilitation Medicine, Department of Orthopedics, Zhejiang Provincial People's Hospital (Affiliated People's Hospital) Hangzhou Medical College, Hangzhou, Zhejiang Province, China; 2 School of Medicine, Zhejiang University, Hangzhou, Zhejiang Province, China; 3 Stomatology Hospital, School of Stomatology, Zhejiang University School of Medicine, Zhejiang Provincial Clinical Research Center for Oral Diseases, Key Laboratory of Oral Biomedical Research of Zhejiang Province, Cancer Center of Zhejiang University, Hangzhou, Zhejiang Province, China

## Abstract

**Background:**

Needlestick injuries (NSIs) are recognised as a major occupational health concern for health care workers, particularly dental assistants who frequently handle sharp instruments. However, little attention has been paid to this vulnerable group. Therefore, it is important to evaluate the prevalence of NSIs among dental assistants and identify associated risk factors to promote the safety level of dental assistants and the development of prevention strategies.

**Methods:**

We followed PRISMA guidelines and registered the protocol with PROSPERO (CRD 42023404766), searching PubMed, Web of Science, Embase, Scopus, and Ovid databases for studies published between January 2000 and January 2023. We selected relevant studies by screening titles and abstracts, and then evaluated the full texts. Risk of bias was assessed using the Critical Appraisal Checklist for prevalence studies proposed by the Joanna Briggs Institute (JBI).

**Results:**

Eleven studies involving 2663 dental assistants were included. The pooled NSIs prevalence was 44% (95% confidence interval (CI) = 0.29–0.59), with significant heterogeneity (*I*^2^ = 98.5%). High-risk procedures were cleaning instrument and handling syringes. Subgroup analysis indicated a declining NSI prevalence trend (53 to 34%). In terms of the degree of national development, NSI prevalence was higher in developed countries (47%) than in developing countries (36%).

**Conclusions:**

The 44% prevalence of NSIs among dental assistants implies a non-negligible risk. Instrument cleaning and the handling of local anaesthetic syringes are the principal procedures and associated factors contributing to their NSI exposure.

**Registration:**

International Prospective Register of Systematic Reviews (CRD 42023404766).

Needlestick injuries (NSIs) is defined as the penetration of skin and mucosal tissue by sharp instruments or needles [1,2]. Needlestick and sharps injuries (NSIs) are recognised as a significant occupational health issue for health care workers globally [3–5] and may lead to transmission of blood-borne pathogens, including hepatitis B virus (HBV), hepatitis C virus (HCV), and human immunodeficiency virus (HIV) [6,7]. Research suggests that NSIs account for 39% of HCV, 37% of HBV, and 4.4% of HIV infections [8]. Additionally, Needlestick injuries (NSIs) can transmit a range of pathogens such as viruses, bacteria, and fungi, inclusive of blastomycosis, herpes, malaria, and various bacterial infections [9].

According to a meta-analysis, the yearly incidence of percutaneous injuries is the highest among health care workers in dental settings in Asia at a rate of 54.8% [10]. Clearly, dental personnel are at greater risk of acquiring occupational infections in comparison to other health care workers [11]. NSI risks among dentists and dental students are well recognised, with a multitude of studies exploring this theme [12–15]. In addition, two systematic reviews and meta-analyses determined the prevalence of NSI among dentists and dental students [16,17]. However, dental assistants, a significant group within dental staff, have not been studied as extensively. When cleaning dental instruments after use, dental assistants are required to handle a large number of contaminated sharp instruments such as dental burrs and dental probes, and do so much more frequently than dentists or students. Dentists focus on diagnosis and treatment, and students have limited and supervised participation. In a survey from four US dental teaching clinics conducted by Ramos-Gomez et al. [18], dental assistants were found to have an exposure incidence density rate six times higher than dental students. Furthermore, most studies of NSI among dental staff have shown underreporting [19]. Given the circumstances, dental assistants' NSI exposure may be significantly underestimated.

The scarcity of reliable prevalence data concerning NSIs among dental assistants impedes the formulation and assessment of preventive strategies. Evidently, comprehending the prevalence of NSIs among dental assistants holds significance for global occupational health policies, especially in low-resource settings. In settings with limited resources, a marginal increase in NSI occurrence can have a more negative effect, potentially leading to the spread of bloodborne pathogens, putting pressure on local health care systems, and causing economic losses. Accurate estimations of NSI prevalence and associated risk factors within this cohort are crucial for formulating effective preventive actions. Overall, this systematic review and meta-analysis aims to determine the prevalence of NSIs among dental assistants, identify related risk factors, heighten their safety awareness, and foster the evolution of preventive measures to enhance the protection of dental assistants globally.

## METHODS

The literature search, study selection, data extraction, and reporting were conducted following the Preferred Reporting Items for Systematic Reviews and Meta-Analysis guidelines (Table S1 in the **Online Supplementary Document**) [20], and the review protocol was registered on International Prospective Register of Systematic Reviews (CRD 42023404766).

### Search strategy

We performed a comprehensive search of PubMed, Web of Science, Embase, Scopus, and Ovid databases for studies published between January 2000 and January 2023. Controlled vocabulary and Boolean operators were used to construct a detailed search strategy. The search terms were: (dental* OR stomatolog* OR oral*) AND (assistant* OR nurse* OR registered nurse* OR practical nurse* OR staff nurse) AND (needlestick injur* OR sharps injur* OR percutaneous exposure* OR occupational injur* OR occupational exposure OR accidental exposure) AND (prevalence* OR rate* OR risk* OR factor*). Additionally, we manually reviewed the reference lists of included articles to identify any further pertinent studies. We endeavoured to make contact with the authors of several potentially relevant studies. Detailed inquiries were dispatched to them, wherein the scope and significance of our systematic review and meta-analysis were elucidated, and requests for additional data from relevant studies were put forward. Regrettably, no responses were received.

### Eligibility criteria

This review focused on articles aligned with the PECO framework. The inclusion criteria were: P) dental assistants; E) needlestick injuries (NSIs); C) not applicable; O) NSI prevalence in dental assistants. We included articles addressing the prevalence of NSIs among dental assistants within a broader dental staff context, including dentists and dental students. However, only the prevalence of NSIs in the dental assistant population was extracted. Only English-language studies were considered. Studies failing to examine or explicitly state the prevalence of NSIs in dental assistants were excluded.

### Study selection and data extraction

Study selection was executed in the following two phases. Two reviewers (JZ and EHL) independently screened titles and abstracts of all selected references according to the predetermined eligibility criteria. In phase two, the authors applied the eligibility criteria to assess the full-text selection of the chosen articles. Discrepancies between the two researchers were addressed through discussion until a consensus was reached. If necessary, a third author (XFY) was consulted to make a final decision when needed.

Data were collected by two researchers (YWL and YX) independently. The information extracted was as follows:

i) article characteristics: first author, year of publication, country, and study design;

ii) sample characteristics: response rate, occurrence (total sample size), and period considered;

iii) main findings: prevalence of NSIs in dental assistants, reporting rate, HBV vaccination, and related factors.

Data extracted from the selected literature underwent a rigorous cross-verification process to assure data accuracy before pooling for analysis.

### Risk of bias for included studies

The same reviewers (JZ and EHL) independently assessed the quality of the eligible studies using a checklist based on a critical appraisal checklist for prevalence studies proposed by the Joanna Briggs Institute (JBI) [21]. The critical appraisal instrument comprises nine distinct components, and the evaluation of each item is recorded as either ‘yes’, ‘no’, ‘unclear’, or ‘not applicable’ (Table S2 in the **Online Supplementary Document**). Any discrepancies were resolved by the discussion method. If there was no agreement between the two reviewers on the final critical appraisal, a third reviewer (XFY) would provide the final opinion on that article. To account for the moderate-risk articles in the analysis, we employed a sensitivity analysis approach.

### Data analysis

We carried out a meta-analysis to estimate the prevalence of NSIs among dental assistants, calculating 95% confidence interval (CI) and using forest plots for visualisation. Study heterogeneity was assessed using the *I*^2^ index with an *I*^2^ over 50% indicating substantial heterogeneity. Accordingly, a random effects model was applied. Subgroup analyses and leave-one-out sensitivity analyses were performed to investigate the sources of heterogeneity [22]. Publication bias was evaluated with Egger's test [23]. Analyses were performed using Stata 16 (Stata Corp. LLC, College Station, USA) and Review Manager 5.4 (The Nordic Cochrane Centre, Copenhagen, Denmark), with 5% marking the statistical significance threshold.

## RESULTS

### Study selection

**Figure 1:**
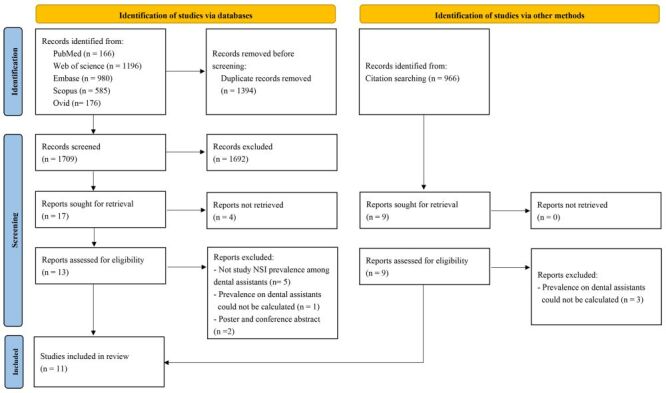
PRISMA flow diagram of the literature search, screening, and selection criteria.

An initial search across various databases yielded 3103 articles, from which 1709 articles remained after removing duplicates for title and abstract screening. Subsequently, 1692 articles were excluded based on title and abstract screening, leaving 17 articles for full-text evaluation. After applying eligibility criteria, 12 articles were excluded. Through manual searches, we identified six additional articles, culminating in 11 studies included for review. The identification, screening, selection, and inclusion processes are delineated in **Figure 1**.

### Study characteristics

**Table 1** displays the characteristics of the included studies. The sample sizes of the 11 selected studies [24–34] varied, ranging from 17 [32] to 894 participants [31]. All articles were observational descriptive studies: eight cross-sectional [25–29,32–34] and three retrospective [24,30,31]. Six studies were published between 2000 and 2011 [29–34]. Geographic distribution included studies from Asia [25,27,34], Europe [28,29,33], North America [30–32], and Oceania [24,26], with seven conducted in developed countries [24,26,28–31,33] and four in developing countries [25,27,32,34]. Six studies reported response rates above 80% [25,27,29,32–34], with one reaching 100% [32]. One study indicated a notably low response rate of 34% [28], and three did not report response rates [24,30,31].

**Table 1:** Detailed characteristics of the included studies

First author	Year	Country/region	Design	Response rate	Occurrence (total sample size)	Period	NSI prevalence	Report rate	HBV vaccination	Procedures involved	Instruments involved	Associated factors
Zachar [24]	2022	Australia	RS	NR	95 (308)	6 y	30.8%	NR	NR	Restorative procedures 46.4%, local anaesthesia 22.1%, and oral surgery 12.3%	Needle 21.8%, probe 20.5%, and burr 18.2%	Repeated capping, uncapping, and disposal of the needle; carelessness; cleaning instruments
Lama [25]	2019	Saudi Arabia	CS	90%	134 (450)	NR	29.8%	36%	85.8%	Recapping 23%	Needles 53%	HBV vaccination; patients in a day; poor knowledge of infection control and disease transmission process; lack of control unit
Siddiqi [26]	2017	New Zealand	CS	66.8%	8 (33)	1 y	24.2%	67.2%	91.6%	Needle recapping	Dental anaesthetic needle 34.8%, burr 28.1%, and scalar tip 21.3%	Lapse in concentration 31.3%, time constraints 14.1%, stress 14.1%, and, fatigue 10.1%
Ebrahimi [27]	2013	Iran	CS	90.2%	70 (228)	1 y	30.7%	NR	76.3%	Washing the instruments, taking the instruments from the tray, or arranging them in the tray	Needle 27.8%, and burr 16.7%	Low practice
van Wijk [28]	2012	Netherlands	CS	34%	101 (198)	1 y	51.2%	NR	88%	Administering anaesthetics	Anaesthetic needles (46%)	Administering anaesthetics and cleaning-up
Wicker [29]	2010	Germany	CS	93.3%	43 (60)	Professional life	71.7%	28.5%	75.4%	Surgical intervention is 55.6% and the instruments used	Surgical devices 46.2%, needles 25.4%, and scalpels 14.2%	Experience; stress 50%, lapses in concentration and fatigue 32.9%
Cleveland [30]	2007	America	RS	NR	80 (360)	10 y	22%	NR	NR	After use of an anaesthetic syringe 60%; use of a dental device 57%; suturing 13%, manipulating a needle in the patient 10%, and handling instruments 9%	Hollow-bore needle 31%, suture needle 15%, burr 10%, scalpel 7%	Cleanup, dismantling, or disposal of dental syringes in the selection
Shah [31]	2006	America	RS	NR	667 (894)	7 y	75%	NR	94%	Cleaning instruments and trays, changing a local anaesthetic carpule 19%, and recapping a needle 18%	Syringe needle 86%, dental instrument (burr, explorer, scaler or scalpel) 9%, suture needle 3%	Cleaning instruments, changing the anaesthetic carpule, and recapping
Smith [32]	2006	West Indies	CS	100%	6 (17)	1 y	35%	100%	100%	Cleaning instrument 60%	Needle 66%; endodontic file 33%	After treatment or shortly after 12 noon; cleaning instrument
Stewardson [33]	2003	The UK	CS	94%	41 (77)	Lifetime; six months	53%, 14%	95%	NR	Handling of the local anaesthetic syringe; cleaning of used instruments	Anaesthetic syringe	Time (afternoon, weekend); fatigue; handling local anaesthetic syringes
Paul [34]	2000	Saudi Arabia	CS	82.2%	22 (38)	1 y	57.9%	41%	47.4%	Clearing the surgery and cleaning instruments	NR	Cleaning instrument; removal of needles and burr after use

CS – cross-sectional study, DA – dental assistant, NR – not reported, NSI – needlestick injury, RS – retrospective study

In this review, 2663 dental assistants were included, with a study of NSI prevalence covering a variety of time frames. Two studies surveyed NSIs over dental assistants' entire careers [29,33], five over one year [26–28,32,34], one over six years [24], one over seven [31], and one study did not specify [30], and one study was not reported [25]. Six studies reported NSI incidence rates [25,26,29,32–34], and eight imparted data on hepatitis B vaccination rates [24,27,28,30,31]. Additionally, eight studies provided information on hepatitis B virus (HBV) vaccination rates [25–29,31,32,34]. The studies detailed the involved procedures, instruments, and risk factors for NSIs.

### The risk of bias in the included studies

**Figure 2:**
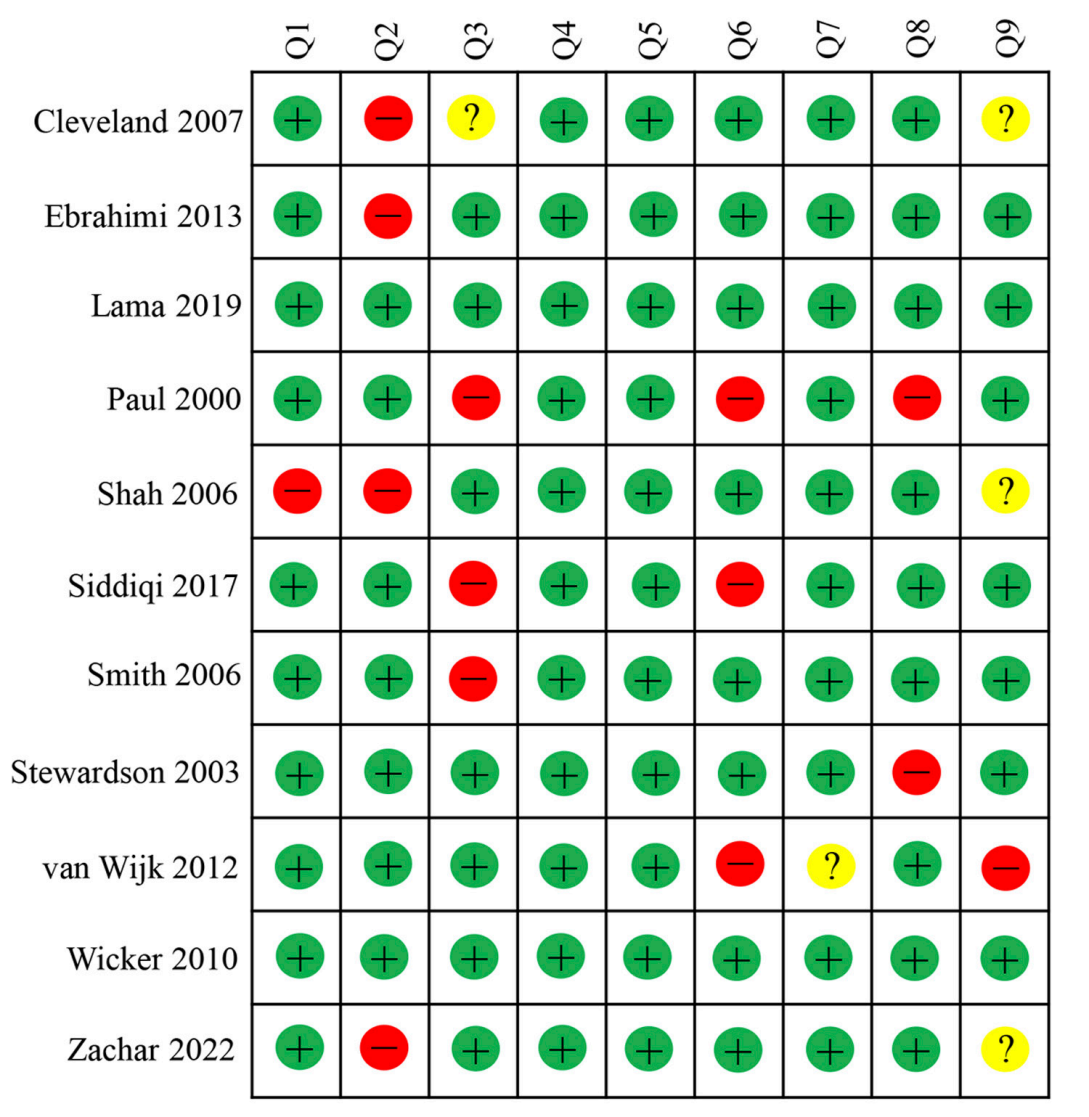
Risk of bias assessment by the Joanna Briggs Institute checklist for prevalence studies. Q1: Was the sample frame appropriate to address the target population? Q2: Were study participants sampled in an appropriate way? Q3: Was the sample size adequate? Q4: Were the study subjects and the setting described in detail? Q5: Was the data analysis conducted with sufficient coverage of the identified sample? Q6: Were valid methods used for the identification of the condition? Q7: Was the condition measured in a standard, reliable way for all participants Q8: Was there appropriate statistical analysis? Q9: Was the response rate adequate, and if not, was the low response rate managed appropriately? + = yes; - = no; ? = unclear.

A quality assessment of the 11 articles identified seven studies with a low risk of bias [24–27,29,32,33], while four presented a moderate risk [28,30,31,34] (**Figure 2**; Table S3 in the **Online Supplementary Document**). Evaluating the risk of bias reflects not only the quality of the literature but also the evidence level of the systematic reviews/meta-analyses.

### Prevalence of dental assistants' NSI

**Figure 3:**
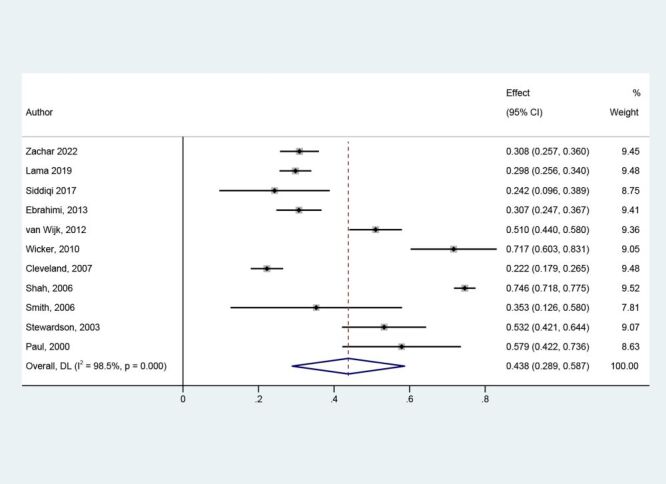
Pooled prevalence among dental assistants. CI – confidence interval.

The NSI prevalence among dental assistants varied notably by country, from 14% in the UK [33] to 75% in the USA [31]. Using a random effects model, the pooled NSI prevalence was 44% (95% CI = 0.29–0.59) (**Figure 3**). Given the substantial heterogeneity (*I*^2^ = 98.5%), subgroup and sensitivity analyses were conducted to explore heterogeneity sources.

### Subgroup analyses and sensitivity analyses

Subgroup analyses were conducted according to the publication year, periods of NSI exposure, development degree of country, and continents (**Table 2**; Figure S1 in the **Online Supplementary Document**). Publication year was chosen to track potential advancements in dental safety and reveal NSI trends and standard impacts. Geographic region is relevant due to its diverse health care, regulations, cultural safety aspects, equipment/training access, and infectious disease differences affecting NSI risks. Development status was taken into account as it relates to occupational health resources, causing prevalence and factor discrepancies between developed and other regions. The analysis by publication year showed NSI prevalence among dental assistants was 53% (95% CI = 0.27–0.78) from 2000 to 2011 and 34% (95% CI = 0.26–0.42) from 2012 to 2023. Regarding timeframes, one-year NSI prevalence was 40% (95% CI = 0.27–0.53), while prevalence during a professional career was 62% (95% CI = 0.44–0.80). At the nation's development level, NSI prevalence was 47% (95% CI = 0.26–0.67) in developed countries and 36% (95% CI = 0.27–0.44) in developing countries. Geographically, NSI prevalence was 30% in Oceania (95% CI = 0.25–0.35), 36% in Asia (95% CI = 0.26–0.46), 58% in Europe (95% CI = 0.46–0.70), and 44% in North America (95% CI = 0.02–0.86) (Figure S2 in the **Online Supplementary Document**).

**Table 2:** Subgroup analysis of the prevalence of NSI in dental assistants

Variable	No. of articles	No. of participants	Prevalence (95% CI)	*I*^2^, %	*P*-value*	Subgroup difference
Prevalence of NSI in different publication years						
*2000–2011 [* *29* *–* *34* *]*	6	1446	53 (0.27–0.78)	99	<0.01	<0.01
*2012–2023 [* *24* *–* *28* *]*	5	1217	34 (0.26–0.42)	87	<0.01	
Prevalence of NSI in different periods						
*1 y prevalence of NSI [* *26* *–* *28* *,* *32* *,* *34* *]*	5	514	40 (0.27–0.53)	86	<0.01	<0.01
*Prevalence of lifetime NSI [* *29* *,* *33* *]*	2	137	62 (0.44–0.80)	80	<0.01	
Prevalence of NSI in different development degree						
*Developed countries [* *24* *,* *26* *,* *28* *–* *31* *,* *33* *]*	7	1210	47 (0.26–0.67)	99	<0.01	<0.01
*Developing countries [* *25* *,* *27* *,* *32* *,* *34* *]*	4	733	36 (0.27–0.44)	74	<0.01	
Prevalence of NSI in different continents						
*Oceania [* *24* *,* *26* *]*	2	128	30 (0.25–0.35)	0	<0.01	<0.01
*Asia [* *25* *,* *27* *,* *34* *]*	3	716	36 (0.26–0.46)	83	<0.01	
*Europe [* *28* *,* *29* *,* *33* *]*	3	335	58 (0.46–0.70)	70	<0.01	
*North America [* *30* *–* *32* *]*	3	764	44 (0.02–0.86)	100	0.04	

CI – confidence interval, NSI – needlestick injury

*Q test.

A sensitivity analysis, excluding four moderate-risk studies [24,25,30,31], produced a consistent pooled NSI prevalence of 39% (95% CI = 0.30,–0.49), albeit with ongoing high heterogeneity (Figure S3 in the **Online Supplementary Document**). Additionally, Egger's test was performed, showing *P* = 0.469 for publication bias, indicating no significant influence on the heterogeneity of the studies (Figure S4 in the **Online Supplementary Document**). Considering the scope of articles and remaining heterogeneity, we retained the original pooled outcome (**Figure 3**).

### Associated procedures and instruments

Among the procedures, eight articles indicated that cleaning instruments was prone to NSIs occurrence [27,30–34], while four articles demonstrated that handling local anaesthetic syringes was likely to result in NSIs [24,28,30,33]. Recapping needles was also a substantial cause [25,26,31]. In addition, NSIs were associated with restorative procedures, oral surgery, and instrument arrangement [24,27,29]. Among the instruments, the syringe needles posed the highest risk [24–28,30–33], followed by burrs, scalpels, and endodontic files [24–26,29–31].

### Associated risk factors

The factors most strongly associated with NSI, as mentioned in the articles included in this study, were cleaning instrument [24,28,30–32,34] and activities related to needle handling, specifically capping, uncapping, and disposal [24,28,30,31,33,34]. Inattention, stress, and fatigue were also linked to NSIs [24,26,29]. Timing influenced NSI occurrences, with higher risk in afternoons and weekends [32,33]. Lack of knowledge, experience, and training contributed to NSI risk [27,29]. One study reported that noncompliance with hepatitis B vaccination protocol and fewer patients were additional factors [25].

## DISCUSSION

Targeted research on needlestick injuries (NSIs) among dental assistants is crucial for enhancing workplace safety and reducing associated psychological and economic burdens. Studies highlight a pronounced variation in reported NSI rates among dental assistants, suggesting a need for targeted research in this group. So, this study holds significant potential to fill existing gaps in the literature. By precisely determining the prevalence and risk factors of NSIs among dental assistants, it lays a solid foundation for the design of highly targeted interventions and safety protocols. Knowledge of the specific prevalence rates and associated risk factors, such as the frequent handling of sharp instruments during cleaning and disposal, enables the customisation of training programmes and the development of safety guidelines that directly address the unique challenges faced by dental assistants. The study of NSIs in dental assistants has a profound impact. Psychologically, dental assistants who experience NSIs often endure significant anxiety about potential infection, which can affect their job satisfaction and overall well-being. This study can raise awareness of this psychological toll and prompt the implementation of support systems to assist affected individuals. Economically, the costs associated with post-exposure prophylaxis and potential medical follow-up are substantial. Understanding the prevalence and risk factors can aid in cost-effective resource allocation. Additionally, it can encourage the dental industry to invest in safer instrument designs and disposal technologies, ultimately reducing the overall economic burden associated with NSIs and enhancing the safety and health of dental assistants.

Accurate assessment of NSI prevalence and associated risks in dental assistants requires improved reporting tools and a deeper understanding of their occupational exposure. The data from the included studies concerning the prevalence of NSIs among dental assistants were gathered using either unvalidated self-designed questionnaires [26,28,29,32–34] or validated questionnaires [25,27]. Indeed, self-reported questionnaires are known to have inherent limitations, particularly in terms of recall bias and underreporting. Despite this limitation, self-reported questionnaires remain a commonly used tool in this field of research due to their practicality and ability to reach a large number of participants. Questionnaires usually report demographic information, details of NSIs, and associated factors. The demographic profile predominantly featured women, with no significant gender-based differences in NSI incidence [29,32]. Two studies confirmed that fingers were the most frequent injury site [24,26], corroborating literature findings [35–37]. Additionally, Zachar and Wicker et al. indicated a higher NSI risk for dental students compared to professionals [24,29]. Conversely, studies such as by van Wijk et al. [28] showed higher reporting rates of NSIs by dental assistants than dentists (51 vs. 24%) OR = 3.23; 95% CI = 1.77–5.90, *P* < 0.001. The cleaning instrument, handling of local anaesthetic syringes, and needle recapping were identified as significant NSI activities [24–34]. Such injuries predominantly involved needles, followed by burrs and scalpels, reflecting their routine work duties. This disparity is likely attributable to the fact that during the disposal process, dental assistants are responsible for sorting and packaging used sharp instruments for subsequent sterilisation or disposal [30]. During this process, they are in frequent contact with potentially infectious sharp objects and may expose themselves to pathogens due to transient lapses in protection, such as unnoticed glove tears. In contrast, dentists and students are less involved in the disposal session, reducing exposure to such risks. Moreover, additional findings reveal that 16% of NSIs pose a high risk for HBV as well as HCV and HIV transmission [28]. Another study found that 8.5% of NSIs were severe and 41.2% occurred when staff were working alone [29]. Collectively, these findings emphasise the importance of not understating NSIs exposure in dental assistants.

Understanding and addressing the diverse risk factors of NSIs are critical for improving workplace safety for dental assistants. Previous systematic reviews have identified NSI prevalence rates of 55.1 among dentists and 44% among dental students [16,17]. Comparable to these findings, our study reveals a 44% NSI prevalence among dental assistants, highlighting their significant risk of NSI. Given the statistics derived from this study, increased vigilance concerning dental assistants' exposure to NSI is warranted. NSI in dental assistants correlates with various risk factors in this study, encompassing cleaning instrument, local anaesthesia procedures (which include disassembly, needle recapping, and disposal), lack of attention, stress, fatigue, timing within the day, clinical experience, patient load, HBV vaccination status, insufficient knowledge of infection control, pathogen transmission, and the absence of a control unit [24–34]. Although the literature presents numerous potential risk factors for NSIs among dental assistants, a consensus has yet to be established. Nevertheless, through our systematic review, among the various potential risk factors, cleaning instrument was identified as the predominant one. The process of cleaning dental instruments is inherently risky as it requires direct handling of sharp and often contaminated items. Dental assistants may be exposed to sharp instrument edges and points during the scrubbing, rinsing, and sterilisation preparation steps due to repetitive motions, and lapses in safety protocols.

Studies also show an association between NSI incidents and the handling of anaesthetic syringes [24,28,30,31,33,34]. When it comes to recapping needles, this task is inherently dangerous as it involves manipulating a sharp object that has already been used. If not done carefully, the needle can easily penetrate the skin. The pressure to complete the task quickly, especially in a busy dental practice, can lead to hasty and improper recapping techniques, thereby elevating the NSI risk. It is similar to the reason dental assistants need to handle post-treatment dental sharp instruments. Factors such as inattention, stress, and fatigue have been found to contribute to NSIs as well [26,29,33]. Stress/fatigue can impair cognitive/motor functions, reducing attention and reaction times. Fatigued assistants may have slow reflexes, and stress can cause distractions, both increasing the risk of instrument mishandling. It is consistent with the causes of NSI caused by high pressure and overwork of dental students [12,17,38,39].

Lack of clinical experience and skills also contributed to the increased risk of NSI [27,29], a trend that also manifests among dentists and dental students [36,38]. NSI incidents are reported to peak following patient treatment, particularly around noon and in the afternoon, possibly attributing to diminished concentration and elevated fatigue levels [32,33]. This pattern aligns with research from China and Brazil [14,15]. Moreover, deficient knowledge of infection control practices has been linked to heightened vulnerability to occupational injuries. Lama et al. [25] found a significant correlation between an absence of infection control knowledge, lack of control units, and increased NSI risk. These findings are mirrored in a Taiwanese study, which observed a 60% increased NSI risk among those uninformed about oral indicators of HIV infection [36]. This underscores the importance of a robust knowledge base regarding infection control and disease transmission in mitigating NSI risk among dental assistants. A study indicated a positive correlation between managing 12 or fewer patients daily and a higher NSI [25]. Conversely, a Chinese study observed a 3.57-fold increase in the risk of injuries from sharp instruments among dentists treating over 30 patients [36]. Although findings are not uniformly consistent, varied risks across populations and geographical areas underscore the need for further investigation into how patient volume affects NSI risk.

Addressing the underreporting of NSIs is essential for effective prevention, resource allocation, and public health strategies. Up to 71.5% of NSI cases of dental assistants remain unreported, and the true exposure of dental assistants to NSI may be substantially underestimated [25,29,34]. At the individual level, underreporting can potentially elevate the risk of infection for both patients and dental personnel. Through the analysis of relevant infection cases, it was found that HBV infection cases could be linked to unreported NSI events, and these unreported events impeded the timely execution of subsequent preventive and treatment measures [7]. From a public health perspective, the underreporting of NSIs will affect the accurate assessment of the epidemic trend of NSIs in the whole region or even the nation, thereby influencing the allocation of resources and the formulation of prevention and control strategies [8]. Underreporting by dental assistants can be attributed primarily to the perceived negligible risk of exposure [26,27,29] and the deterrent effect of heavy workloads coupled with lengthy reporting procedures for NSI report [32]. Therefore, it is evident that enhancing infection control training and streamlining reporting processes could increase the reporting rates, thus facilitating better management of sharp instrument injuries post-exposure. Specific recommendations are as follows: Simplify reporting and reduce time and paperwork through user-friendly digital platforms. Meanwhile, develop training programmes that focus on NSI prevention, report importance/process, use interactive modules, case studies, and review to ensure that dental assistants are well-informed and vigilant.

Declining trends and global disparities in NSI prevalence highlight the need for improved reporting standards and methodological consistency. In our subgroup analysis, the prevalence of NSI among dental assistants decreased from 53 in 2000–2011 to 34% in 2012–2023, which was lower than the overall prevalence of 44% among dental assistants in this study, possibly indicating a downward trend in NSI prevalence due to better training and increased safety awareness. Because the dental field has enhanced protocols like using sharps safety devices, better sterilisation, and more training, which dental institutions and associations have promoted. However, the reason for this result could also be due to the high heterogeneity between studies or methodological differences. Changes in design, sampling, and data collection, such as earlier self-reported data with biases vs later objective measures, and differences in NSI definitions, could affect prevalence. In the future, the study characteristics of the two aspects can be analysed to understand the real reasons for the decreased prevalence in terms of safety/training and methodology and to provide more accurate explanations. Of note, when stratifying by country development status, the prevalence in developed countries (47%) exceeded that in developing countries (36%). The difference may be due to developed nations' better reporting via comprehensive surveillance, while developing countries may underreport due to data collection issues. Occupational safety protocol differences also count. Developed countries have advanced programmes with good equipment, training, and strict rules. Developing countries may have less safety measure implementation because of financial and awareness problems, leading to a potentially higher actual but lower reported prevalence. To understand better, in the future it is necessary to do a more comprehensive study, collecting data on safety protocols and reporting systems and correlating with prevalence factors like equipment, training, and regulation enforcement. Overall, the results of the subgroup analysis showed a trend in the prevalence of NSIs in dental assistants to some extent and highlighted the need for higher quality studies in the future to accurately assess the prevalence of NSI in dental assistants and its influencing factors. The results of subgroup analyses should be considered as ancillary to the primary outcome to avoid incorrect reporting of study results.

The pronounced heterogeneity in this study, which remains unresolved following subgroup and sensitivity analyses, underscores the challenges in synthesising findings across diverse methodologies and participant characteristics. This may be attributed to several factors. First, disparities in study design; the inclusion of diverse study types, such as cross-sectional and retrospective designs, might give rise to heterogeneity. Second, variances in data collection methods; specifically, the utilisation of self-report questionnaires in contrast to direct observation or medical record review might lead to differences. As previously noted, self-reported data are susceptible to recall bias and underreporting. Third, the heterogeneity could stem from participant characteristics; differences in the age, gender, work experience, and training levels of dental assistants across studies may account for the observed variability.

Effectively reducing NSIs among dental assistants necessitates standardised prevention strategies, comprehensive training, and practical, context-specific measures to mitigate their prevalence. However, adherence to adequate precautions remains insufficient [26,29] and requires reinforcement. Research consistently indicates the need for heightened awareness among dental staff regarding exposure injuries and the implementation of standard preventive measures and infection control training to decrease NSI incidents [12,40–43]. Instrument cleaning is a primary source of NSIs for dental assistants. Thus, for high-risk cleaning processes, the utilisation of automatic cleaning devices can effectively reduce the prevalence of dental assistants. For resource-limited settings, rather than relying only on costly automatic cleaning devices, we can consider simple, safety-designed manual cleaning kits with ergonomic tools to cut cleaning injury risks. Also, promoting disposable instruments when feasible can ease the need for elaborate and expensive cleaning and sterilisation. Moreover, studies have confirmed that the immediate removal of burrs from handpieces after treatment, along with the use of burr blocks, cassettes, and disposable probes, substantially reduces NSIs [24]. Handling local anaesthetic syringes is the second major cause of NSIs in dental assistants. Regarding anaesthesia syringes, applying one-handed recapping techniques, adopting a no-recap policy for needles, and utilising safety-engineered syringes contribute to the prevention of NSI [24,26,44]. Simultaneously, to prevent NSIs among dental assistants resulting from diverse factors, it is essential to formulate targeted standardised operating procedures, thereby enabling dental assistants to acquire the requisite knowledge and skills prior to conducting clinical operations [45]. In light of the associated risk factors of NSI among dental assistants identified through this systematic review, the following specific preventive measures are summarised:

a) education and training: national/regional mandatory programmes based on international safety standards like ISO's could ensure standardised knowledge, enhancing infection control training to raise awareness and knowledge amongst dental assistants;

b) improved work practices: policies using WHO's sharps safety guidelines could be incorporated into rules of hospital, such as promote the use of safe sharps handling techniques and the implementation of no-recapping policies;

c) work scheduling: minimise fatigue-related risks by managing schedules and patient load effectively;

d) vaccination and health monitoring: ensure that all employees complete the recommended vaccination, and health monitoring on a regular basis;

e) encourage reporting: introduce simplified and standardised NSI reporting forms and procedures to encourage accurate reporting and post-exposure management.

Several limitations related to this review should be acknowledged. First of all, the exclusion of non-English language publications may introduce publication bias and restrict the analysis of data from various regions or countries, diminishing the global applicability of the results. The exclusion was due to practical issues (limited team language skills and resource few for translation). Future systematic reviews of the prevalence of NSI in dental assistants in specific language areas are needed. Second, although the prevalence of NSI in dental assistants was pooled, there was significant heterogeneity. It is also worth noting that our study did not include data from regions such as Africa or South America, and the number of articles included in the subgroup analyses was small. To address this deficit and enhance global understanding of NSIs, it is critical to conduct future research efforts in South America and Africa. Furthermore, the reliance on self-reported questionnaires for data collection could result in recall bias, potentially underrepresenting the true incidence rate of NSIs. It emphasises the need for caution when interpreting the results obtained from such data sources and suggest that future studies could explore alternative data collection methods or combine multiple approaches to mitigate these biases, such as the use of objective exposure measures and questionnaires with high reliability and validity. Thus, it is acknowledged that the pooled data in the present review may not truly represent the worldwide current prevalence of NSIs among dental assistants. More comprehensive and higher-quality studies are necessary to facilitate more accurate assessments. Despite these constraints, the review substantially contributes to the understanding of NSI prevalence and associated risk factors among dental assistants. The significance of these findings stresses the need for attention to the safety and health of dental assistants. Identifying and analysing the specific procedures, instruments, and risk factors that contribute to NSIs in this group are essential to advancing practice guidelines and fostering educational initiatives to prevent NSIs.

## CONCLUSIONS

The 44% prevalence of NSIs among dental assistants implies a non-negligible risk. Instrument cleaning and the handling of local anaesthetic syringes are the principal procedures and associated factors contributing to their NSI exposure. Notwithstanding its limitations, this study is conducive to understanding NSIs in dental assistants and offers a reference for prevention.

## Additional material


Online Supplementary Document
