# Supplementary material for: Prevalence of needlestick injuries in dental assistants: a systematic review and meta-analysis
Source: J Glob Health. 2025 Feb 14;15:04030. doi: 10.7189/jogh.15.04030 (PMC11826961; doi:10.7189/jogh.15.04030)
Supplement: Online Supplementary Document [file jogh-15-04030-s001.pdf]

Supplementary Figure S1. Subgroup analysis of the prevalence of NSI in dental assistants

Study or Subgroup	Mean Difference	SE	Weight	Mean Difference IV, Random, 95% CI
**5.1.1 2000-2011**				
Cleveland 2007	0.22222222	0.02191141	9.5%	0.22 [0.18, 0.27]
Paul 2000	0.57894737	0.08009325	8.6%	0.58 [0.42, 0.74]
Shah 2006	0.74608501	0.01455692	9.5%	0.75 [0.72, 0.77]
Smith 2006	0.35294118	0.11590404	7.8%	0.35 [0.13, 0.58]
Stewardson 2003	0.53246753	0.05686003	9.1%	0.53 [0.42, 0.64]
Wicker 2010	0.71666667	0.05817439	9.0%	0.72 [0.60, 0.83]
**Subtotal (95% CI)**			**53.6%**	**0.53 [0.27, 0.78]**
Heterogeneity: Tau² = 0.10; Chi² = 406.55, df = 5 (P < 0.00001); I² = 99%				
Test for overall effect: Z = 4.05 (P < 0.0001)				
**5.1.2 2012-2023**				
Ebrahimi 2013	0.30701754	0.03054747	9.4%	0.31 [0.25, 0.37]
Lama 2019	0.29777778	0.02155645	9.5%	0.30 [0.26, 0.34]
Siddiqi 2017	0.24242424	0.0746009	8.7%	0.24 [0.10, 0.39]
van Wijk 2012	0.51010101	0.0355262	9.4%	0.51 [0.44, 0.58]
Zachar 2022	0.30844156	0.02631634	9.4%	0.31 [0.26, 0.36]
**Subtotal (95% CI)**			**46.4%**	**0.34 [0.26, 0.42]**
Heterogeneity: Tau² = 0.01; Chi² = 30.55, df = 4 (P < 0.00001); I² = 87%				
Test for overall effect: Z = 8.52 (P < 0.00001)				
**Total (95% CI)**			**100.0%**	**0.44 [0.29, 0.59]**
Heterogeneity: Tau² = 0.06; Chi² = 649.45, df = 10 (P < 0.00001); I² = 98%				
Test for overall effect: Z = 5.77 (P < 0.00001)				
Test for subgroup differences: Chi² = 1.92, df = 1 (P = 0.17), I² = 47.9%				

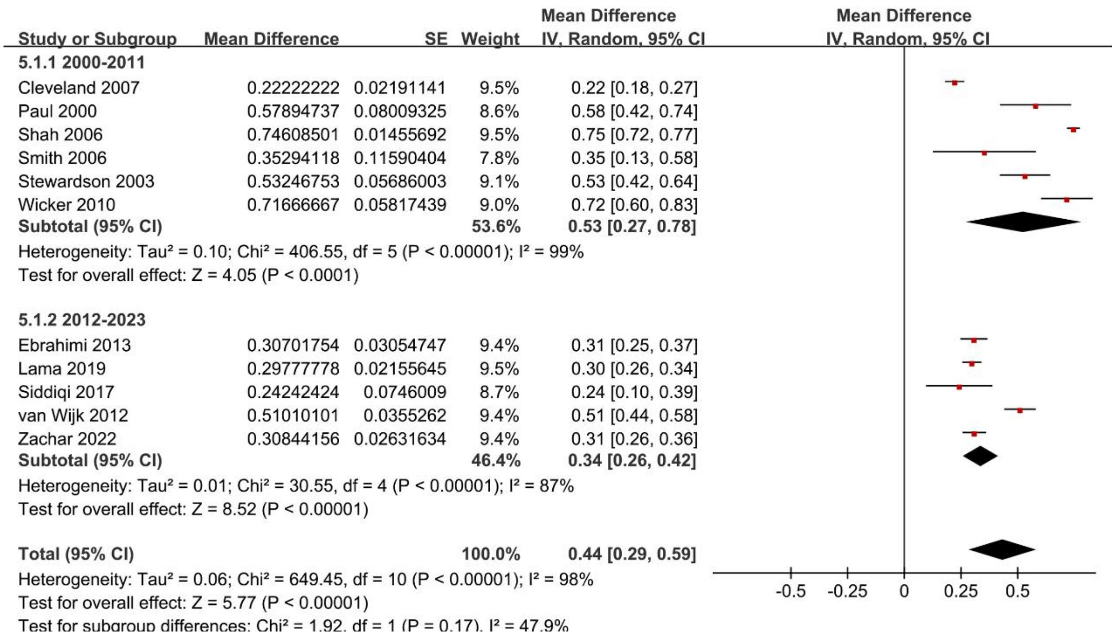

A. Prevalence of dental assistant NSI in different publication year

Study or Subgroup	Mean Difference	SE	Weight	Mean Difference IV, Random, 95% CI
**1.1.1 1 year prevalence of NSI**				
Ebrahimi 2013	0.30701754	0.03054747	16.3%	0.31 [0.25, 0.37]
Paul 2000	0.57894737	0.08009325	13.4%	0.58 [0.42, 0.74]
Siddiqi 2017	0.24242424	0.0746009	13.7%	0.24 [0.10, 0.39]
Smith 2006	0.35294118	0.11590404	10.9%	0.35 [0.13, 0.58]
van Wijk 2012	0.51010101	0.0355262	16.1%	0.51 [0.44, 0.58]
**Subtotal (95% CI)**			**70.3%**	**0.40 [0.27, 0.53]**
Heterogeneity: Tau² = 0.02; Chi² = 28.37, df = 4 (P < 0.0001); I² = 86%				
Test for overall effect: Z = 6.12 (P < 0.00001)				
**1.1.2 Prevalence of lifetime NSI**				
Stewardson 2003	0.53246753	0.05686003	14.9%	0.53 [0.42, 0.64]
Wicker 2010	0.71666667	0.05817439	14.8%	0.72 [0.60, 0.83]
**Subtotal (95% CI)**			**29.7%**	**0.62 [0.44, 0.80]**
Heterogeneity: Tau² = 0.01; Chi² = 5.13, df = 1 (P = 0.02); I² = 80%				
Test for overall effect: Z = 6.78 (P < 0.00001)				
**Total (95% CI)**			**100.0%**	**0.47 [0.34, 0.59]**
Heterogeneity: Tau² = 0.02; Chi² = 58.69, df = 6 (P < 0.00001); I² = 90%				
Test for overall effect: Z = 7.29 (P < 0.00001)				
Test for subgroup differences: Chi² = 3.95, df = 1 (P = 0.05), I² = 74.7%				

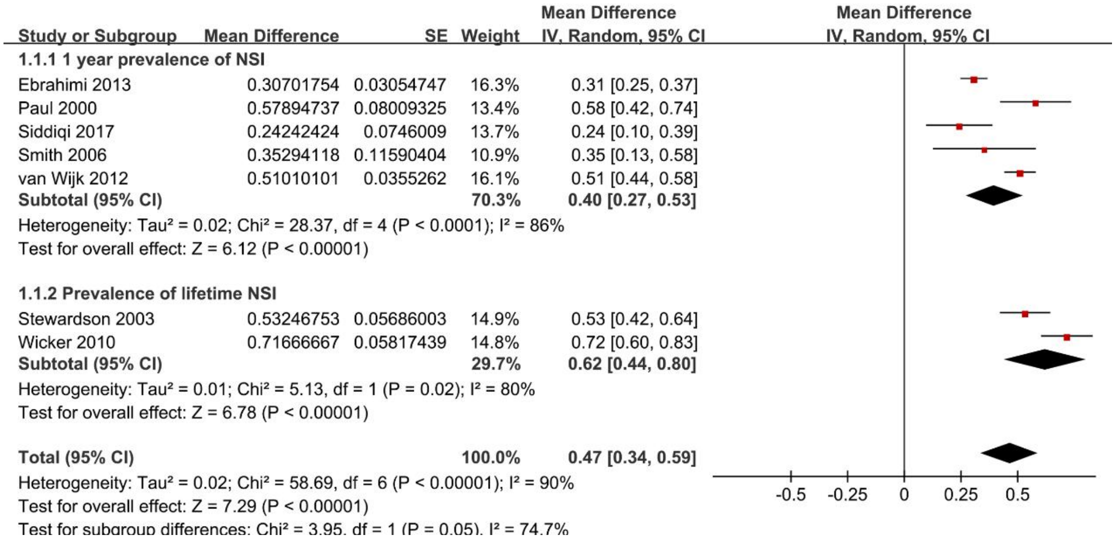

B. Prevalence of dental assistant NSI in time periods

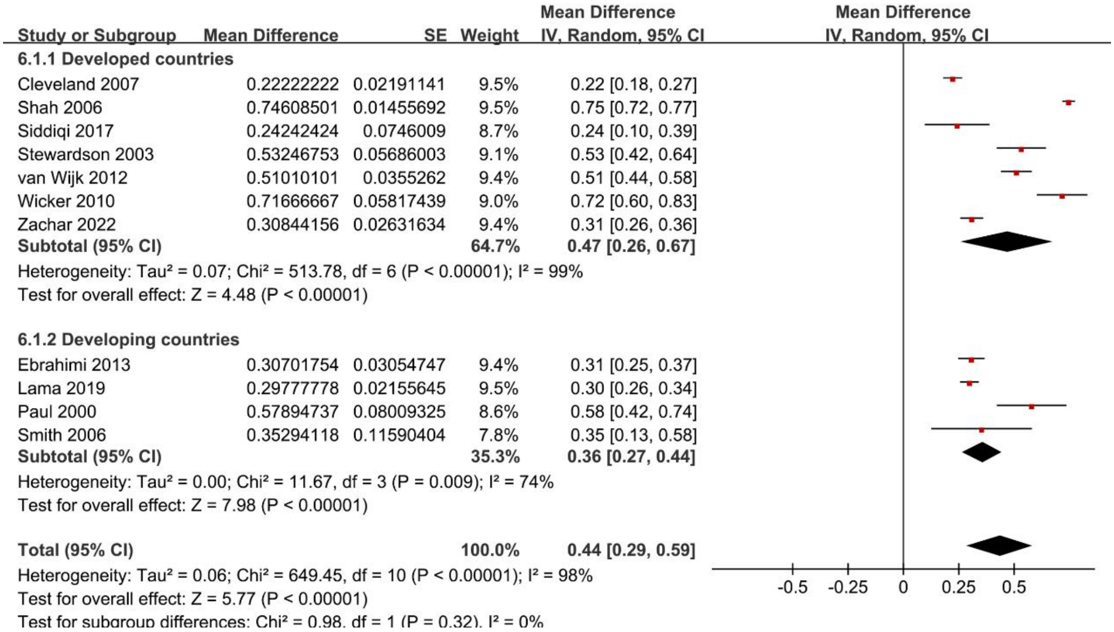

C. Prevalence of dental assistant NSI in different development degree

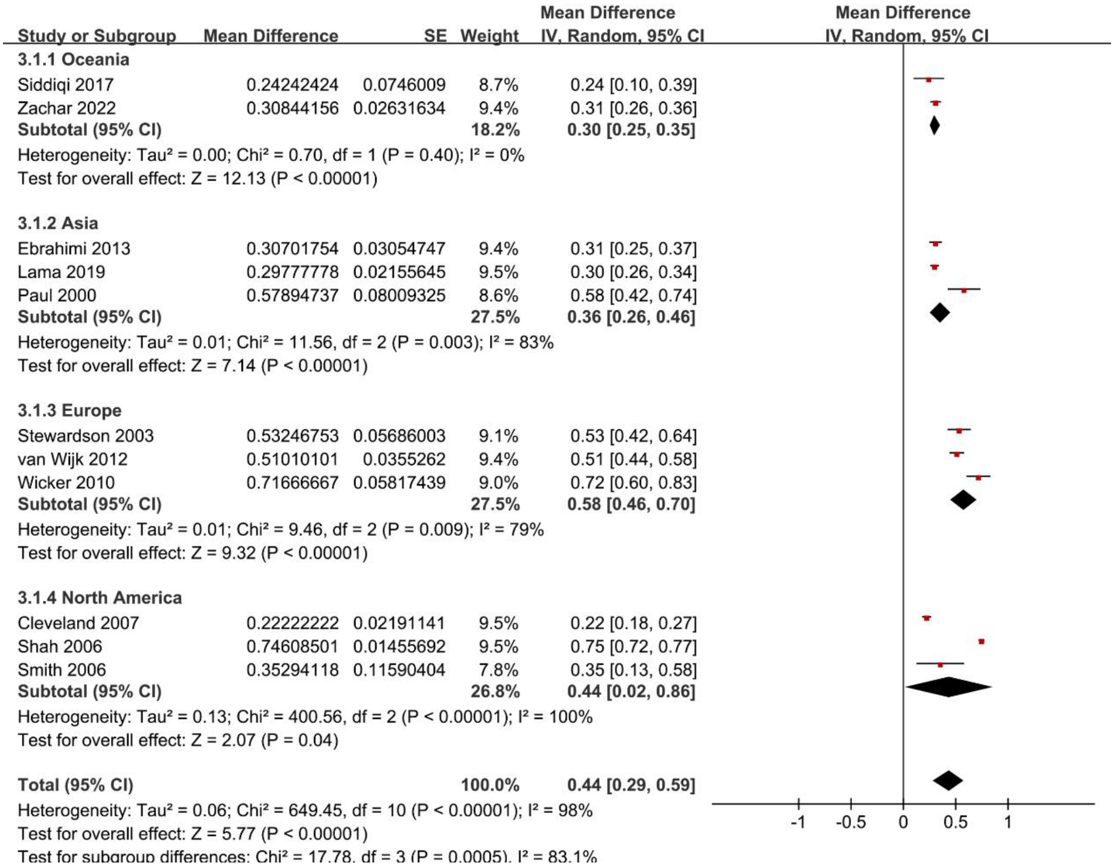

D. Prevalence of dental assistants NSI in different continents

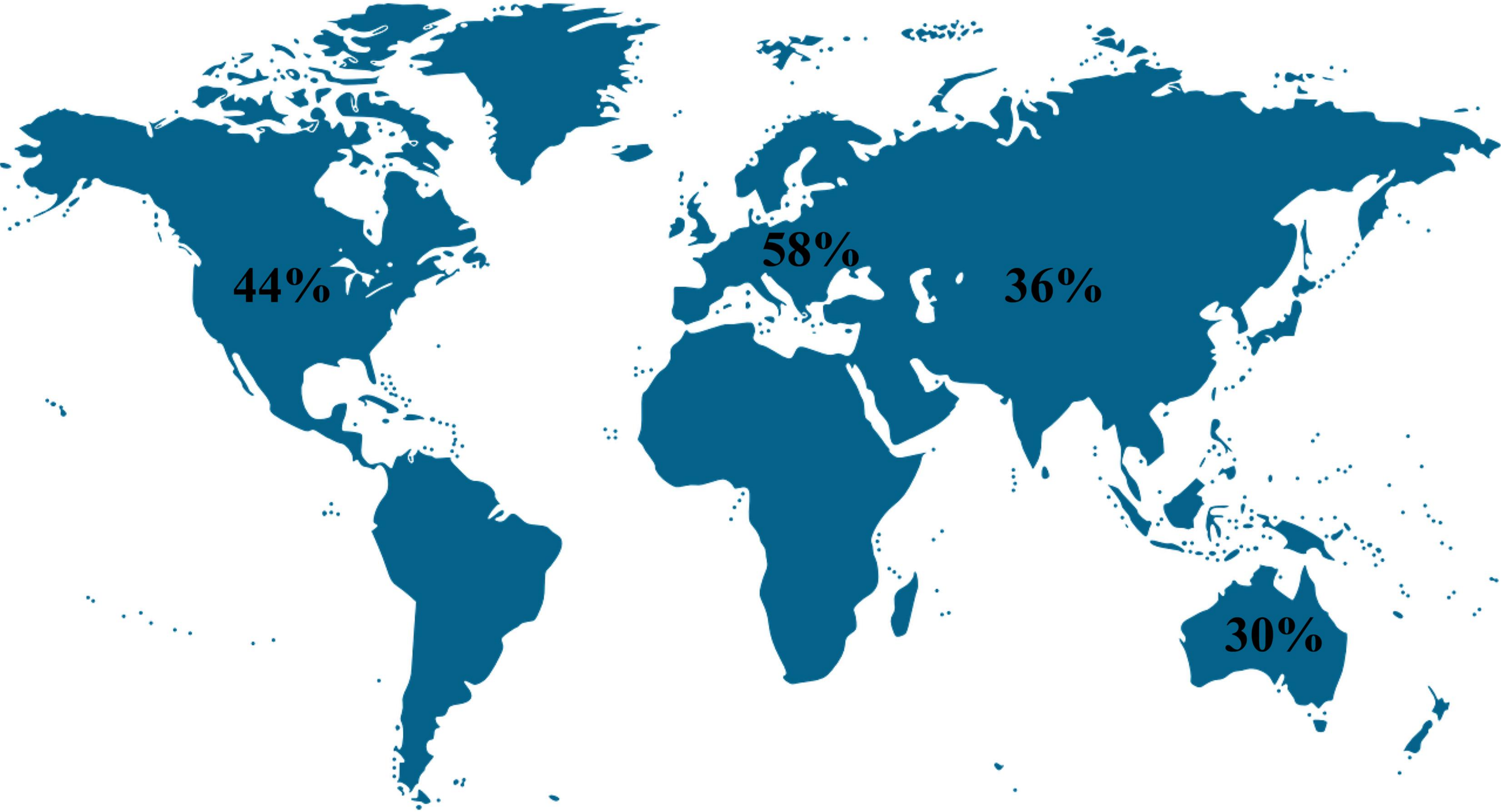
58%
44%
36%
30%

Supplementary Figure S3. Sensitivity analysis in included studies

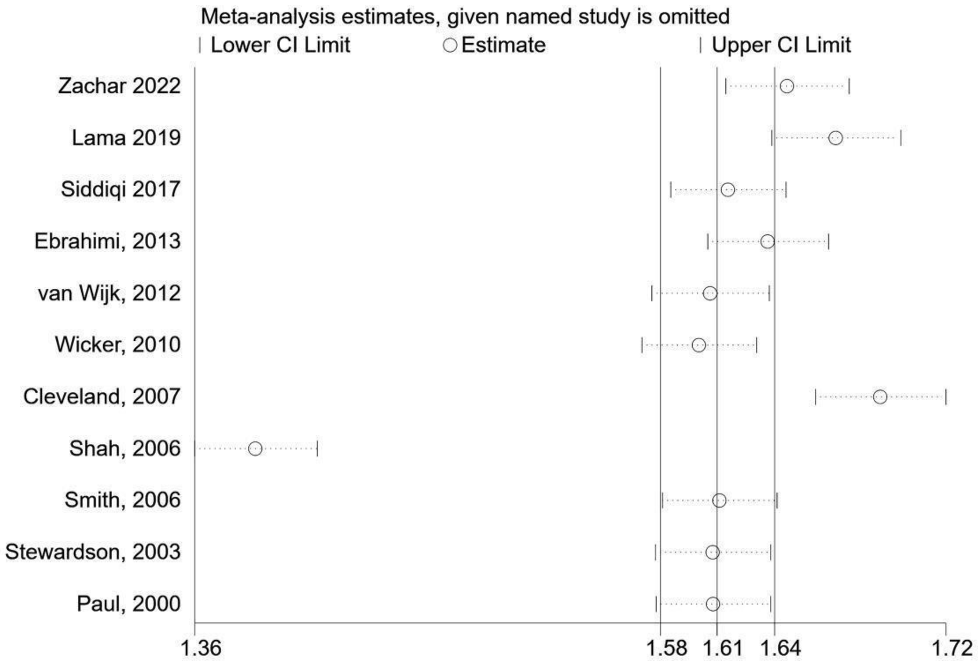

Results of the sensitivity analysis

Study or Subgroup	Mean Difference	SE	Weight	Mean Difference IV, Random, 95% CI
Cleveland 2007	0.22222222	0.02191141	0.0%	0.22 [0.18, 0.27]
Ebrahimi 2013	0.30701754	0.03054747	16.5%	0.31 [0.25, 0.37]
Lama 2019	0.29777778	0.02155645	17.0%	0.30 [0.26, 0.34]
Paul 2000	0.57894737	0.08009325	0.0%	0.58 [0.42, 0.74]
Shah 2006	0.74608501	0.01455692	0.0%	0.75 [0.72, 0.77]
Siddiqi 2017	0.24242424	0.0746009	12.5%	0.24 [0.10, 0.39]
Smith 2006	0.35294118	0.11590404	8.9%	0.35 [0.13, 0.58]
Stewardson 2003	0.53246753	0.05686003	14.2%	0.53 [0.42, 0.64]
van Wijk 2012	0.51010101	0.0355262	0.0%	0.51 [0.44, 0.58]
Wicker 2010	0.71666667	0.05817439	14.1%	0.72 [0.60, 0.83]
Zachar 2022	0.30844156	0.02631634	16.8%	0.31 [0.26, 0.36]
**Total (95% CI)**			**100.0%**	**0.39 [0.30, 0.49]**

Heterogeneity: Tau² = 0.01; Chi² = 61.49, df = 6 (P < 0.00001); I² = 90%
Test for overall effect: Z = 7.99 (P < 0.00001)

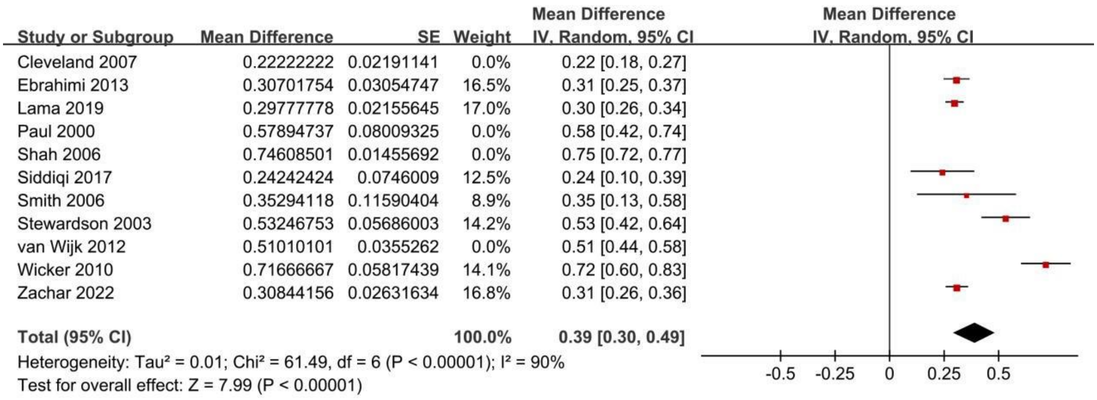

Supplementary Figure S4. Egger' test in included studies

```
Egger's test for small-study effects:
Regress standard normal deviate of intervention
  effect estimate against its standard error

.
Number of studies =   11                          Root MSE      =    8.238
------------------------------------------------------------------------------
     Std_Eff |      Coef.   Std. Err.      t    P>|t|     [95% Conf. Interval]
-------------+----------------------------------------------------------------
       slope |    .568752   .1404034     4.05   0.003     .2511375    .8863664
        bias |  -3.683509   4.877824    -0.76   0.469    -14.71791    7.350896
------------------------------------------------------------------------------

Test of H0: no small-study effects          P = 0.469
```

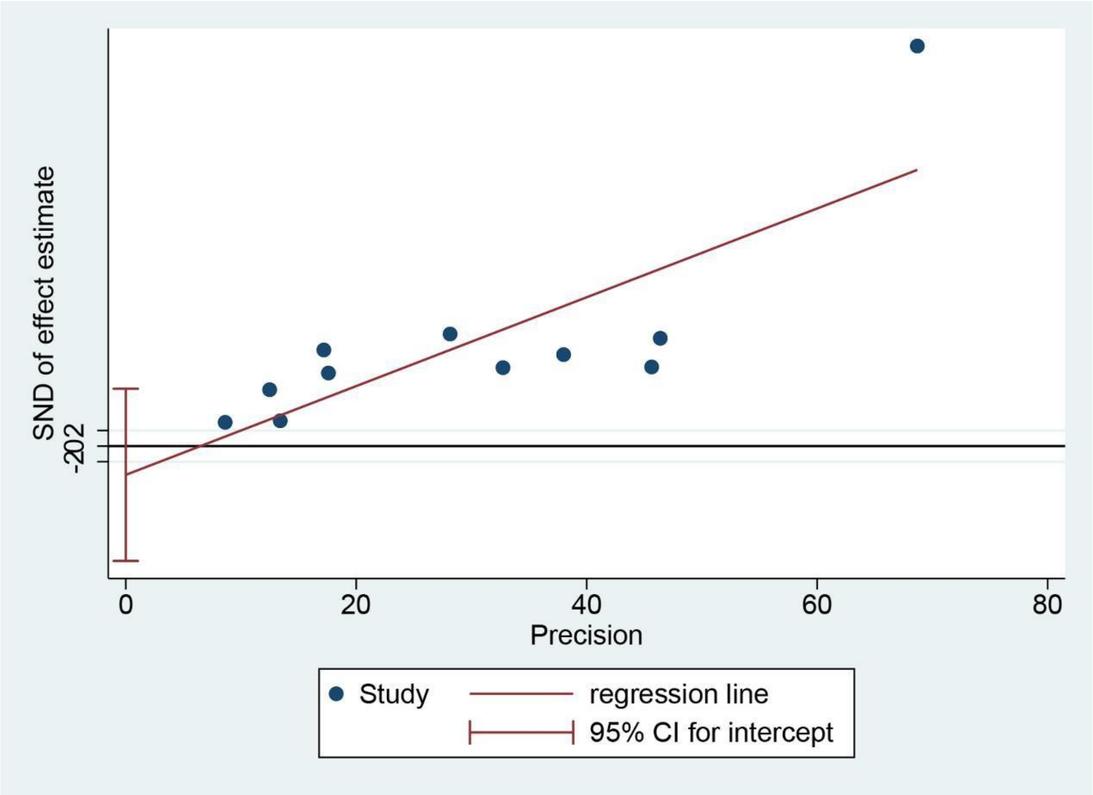

Supplementary Table S1. PRISMA 2020 Checklist

Section and Topic	Item #	Checklist item	Location where item is reported
TITLE			
Title	1	Identify the report as a systematic review.	1
ABSTRACT			
Abstract	2	See the PRISMA 2020 for Abstracts checklist.	2
INTRODUCTION			
Rationale	3	Describe the rationale for the review in the context of existing knowledge.	3
Objectives	4	Provide an explicit statement of the objective(s) or question(s) the review addresses.	4
METHODS			
Eligibility criteria	5	Specify the inclusion and exclusion criteria for the review and how studies were grouped for the syntheses.	5
Information sources	6	Specify all databases, registers, websites, organisations, reference lists and other sources searched or consulted to identify studies. Specify the date when each source was last searched or consulted.	4-5
Search strategy	7	Present the full search strategies for all databases, registers and websites, including any filters and limits used.	4-5
Selection process	8	Specify the methods used to decide whether a study met the inclusion criteria of the review, including how many reviewers screened each record and each report retrieved, whether they worked independently, and if applicable, details of automation tools used in the process.	5
Data collection process	9	Specify the methods used to collect data from reports, including how many reviewers collected data from each report, whether they worked independently, any processes for obtaining or confirming data from study investigators, and if applicable, details of automation tools used in the process.	6
Data items	10a	List and define all outcomes for which data were sought. Specify whether all results that were compatible with each outcome domain in each study were sought (e.g. for all measures, time points, analyses), and if not, the methods	6

Section and Topic	Item #	Checklist item	Location where item is reported
		used to decide which results to collect.	
	10b	List and define all other variables for which data were sought (e.g. participant and intervention characteristics, funding sources). Describe any assumptions made about any missing or unclear information.	
Study risk of bias assessment	11	Specify the methods used to assess risk of bias in the included studies, including details of the tool(s) used, how many reviewers assessed each study and whether they worked independently, and if applicable, details of automation tools used in the process.	6
Effect measures	12	Specify for each outcome the effect measure(s) (e.g. risk ratio, mean difference) used in the synthesis or presentation of results.	6-7
Synthesis methods	13a	Describe the processes used to decide which studies were eligible for each synthesis (e.g. tabulating the study intervention characteristics and comparing against the planned groups for each synthesis (item #5)).	
	13b	Describe any methods required to prepare the data for presentation or synthesis, such as handling of missing summary statistics, or data conversions.	
	13c	Describe any methods used to tabulate or visually display results of individual studies and syntheses.	6
	13d	Describe any methods used to synthesize results and provide a rationale for the choice(s). If meta-analysis was performed, describe the model(s), method(s) to identify the presence and extent of statistical heterogeneity, and software package(s) used.	7
	13e	Describe any methods used to explore possible causes of heterogeneity among study results (e.g. subgroup analysis, meta-regression).	7
	13f	Describe any sensitivity analyses conducted to assess robustness of the synthesized results.	7
Reporting bias assessment	14	Describe any methods used to assess risk of bias due to missing results in a synthesis (arising from reporting biases).	6
Certainty	15	Describe any methods used to assess certainty (or confidence) in the body of evidence for an outcome.	6

Section and Topic	Item #	Checklist item	Location where item is reported
assessment			
RESULTS			
Study selection	16a	Describe the results of the search and selection process, from the number of records identified in the search to the number of studies included in the review, ideally using a flow diagram.	7
	16b	Cite studies that might appear to meet the inclusion criteria, but which were excluded, and explain why they were excluded.	Figure 1
Study characteristic s	17	Cite each included study and present its characteristics.	7-8
Risk of bias in studies	18	Present assessments of risk of bias for each included study.	8
Results of individual studies	19	For all outcomes, present, for each study: (a) summary statistics for each group (where appropriate) and (b) an effect estimate and its precision (e.g. confidence/credible interval), ideally using structured tables or plots.	Table 1
Results of syntheses	20a	For each synthesis, briefly summarise the characteristics and risk of bias among contributing studies.	9
	20b	Present results of all statistical syntheses conducted. If meta-analysis was done, present for each the summary estimate and its precision (e.g. confidence/credible interval) and measures of statistical heterogeneity. If comparing groups, describe the direction of the effect.	9-10
	20c	Present results of all investigations of possible causes of heterogeneity among study results.	10
	20d	Present results of all sensitivity analyses conducted to assess the robustness of the synthesized results.	9-10
Reporting biases	21	Present assessments of risk of bias due to missing results (arising from reporting biases) for each synthesis assessed.	8
Certainty of	22	Present assessments of certainty (or confidence) in the body of evidence for each outcome assessed.	Figure 3

Section and Topic	Item #	Checklist item	Location where item is reported
evidence			

Supplementary Table S2. JBI critical appraisal checklist for studies reporting prevalence data

Reviewer ______________________________________

Date________________________________

Author____ Year_________ Record Number_________

	Yes	No	Unclear	Not applicable
Was the sample frame appropriate to address the target population?	□	□	□	□
Were study participants sampled in an appropriate way?	□	□	□	□
Was the sample size adequate?	□	□	□	□
Were the study subjects and the setting described in detail?	□	□	□	□
Was the data analysis conducted with sufficient coverage of the identified sample?	□	□	□	□
Were valid methods used for the identification of the condition?	□	□	□	□
Was the condition measured in a standard, reliable way for all participants?	□	□	□	□
Was there appropriate statistical analysis?	□	□	□	□
Was the response rate adequate, and if not, was the low response rate managed appropriately?	□	□	□	□

Overall appraisal: Include □ Exclude □ Seek further info □

Comments (Including reason for exclusion)

____

____

____

Supplementary Table S3. Risk of bias assessment in individual studies

Author, year	Q1	Q2	Q3	Q4	Q5	Q6	Q7	Q8	Q9	Risk of bias
Zachar, 2022	Y	N	Y	Y	Y	Y	Y	Y	UN	Low
Lama, 2019	Y	Y	Y	Y	Y	Y	Y	Y	Y	Low
Siddiqi, 2017	Y	Y	N	Y	Y	N	Y	Y	Y	Low
Ebrahimi, 2013	Y	N	Y	Y	Y	Y	Y	Y	Y	Low
van Wijk, 2012	Y	Y	Y	Y	Y	N	UN	Y	N	Moderate
Wicker, 2010	Y	Y	Y	Y	Y	Y	Y	Y	Y	Low
Cleveland, 2007	Y	N	UN	Y	Y	Y	Y	Y	UN	Moderate
Shah, 2006	N	N	Y	Y	Y	Y	Y	Y	UN	Moderate
Smith, 2006	Y	Y	N	Y	Y	Y	Y	Y	Y	Low
Stewardson, 2003	Y	Y	Y	Y	Y	Y	Y	N	Y	Low
Paul, 2000	Y	Y	N	Y	Y	N	Y	N	Y	Moderate

Legend: Y= Yes; N= No; UN= Unclear

Q1- Was the sample frame appropriate to address the target population?

Q2- Were study participants sampled in an appropriate way?

Q3- Was the sample size adequate?

Q4- Were the study subjects and the setting described in detail?

Q5- Was the data analysis conducted with sufficient coverage of the identified sample?

Q6- Were valid methods used for the identification of the condition?

Q7- Was the condition measured in a standard, reliable way for all participants?

Q8- Was there appropriate statistical analysis?

Q9- Was the response rate adequate, and if not, was the low response rate managed appropriately?

The studies were assigned high, moderate, or low risk of bias if they fulfilled 3, 4-6, and 7-9 criteria, respectively.
